# Dental Suggestions

**Published:** 1885-06

**Authors:** John D. Wingate

**Affiliations:** Carbondale, Pa.


### DENTAL SUGGESTIONS.

BY DR. JOHN D. WINGATE, CARBONDALE, PA.

### AMALGAM.

Is there not some danger that our younger brethren, who are ardent pursuers of the best modes of serving their patrons, may fall into grievous errors from the writings and sayings of some of our distinguished lights in the profession? Among the noted errors taught is that of using homœopathic quantities of mercury in the preparation of amalgam. There is a proper quantity to form a perfect combination of the material, and less gives a brittle, pasty stuff, unfit to remain in the mouth. A properly prepared amalgam filling, in a well prepared cavity, will preserve that part of the tooth.

### PLASTIC FILLINGS.

The market at present is groaning under a plethora of plastic filling material, much of which is only a fraud, and it should be our duty to freeze out the worthless preparations. High sounding names and questionable recommendations do not change the worthless into good.

TESTING FILLINGS.

A practitioner who wears a rubber or a celluloid plate has one advantage over those who have only natural teeth. Cavities may be drilled into the plates, and fillings tested. Two classes of cavities may be made; one where friction will act on the fillings, and another not exposed to friction, but where the fluids of the mouth may act as solvents. The old and the new may then be tested side by side, and the most eccentric conditions complied with.

REFITTING LOWER PLATES.

When absorption has spoiled the fit or injured the articulation, the old plate may be filled with plaster of paris, prepared as for taking an impression, and carried to its place, directing the patient to close the upper teeth upon it. This gives the impression and the "bite" at the same time. Then the cast may be made and connected with the lower part of the articulating model, while the upper part may have half the crowns of the teeth inserted into its part of the plaster. In this way the bite is retained, no matter how much it may be lengthened.

ANNEALING GOLD FOIL.

Much time is lost by passing the gold, during filling, through the flame. A piece of Russia sheet-iron, six inches square, will serve a good purpose. The gold, properly prepared, may be spread on this and placed on any heating apparatus. The heat need not exceed 700 degrees, or the melting point of lead, and it may frequently be repeated at that point and still work well.

